# Discrimination of *Polygonatum* Species via Polysaccharide Fingerprinting: Integrating Their Chemometrics, Antioxidant Activity, and Potential as Functional Foods

**DOI:** 10.3390/foods14132385

**Published:** 2025-07-05

**Authors:** Zhiguo Liu, Wei Zhang, Bin Wang

**Affiliations:** 1 Key Laboratory of Xin’an Medicine of the Ministry of Education, College of Chinese Medicine, School of Pharmacy, and Functional Activity and Resource Utilization on Edible and Medicinal Fungi Joint Laboratory of Anhui Province, Anhui University of Chinese Medicine, Hefei 230038, China; 19355976019@163.com; 2 Anhui Province Key Laboratory of Traditional Chinese Medicine Decoction Pieces of New Manufacturing Technology, Hefei 230012, China

**Keywords:** *Polygonati* Rhizoma, polysaccharide, functional food ingredient, fingerprint identification, PCA, HCA, OPLS-DA, antioxidant activity

## Abstract

*Polygonati* Rhizoma, a renowned edible homologous material, encompasses an array of widely distributed species. Despite their morphological and medicinal similarities, their overlapping distribution and evolving varieties present challenges for their classification and identification. This study provides a comprehensive characterization of the physicochemical and antioxidant properties of polysaccharides extracted from three common species: *P. sibiricum*, *P. cyrtonema*, and *P. kingianum*. An analysis of their monosaccharide composition reveals distinct profiles, with *P. kingianum* polysaccharides (PKPs) demonstrating a significantly higher glucose content compared to *P. sibiricum* polysaccharides (PSPs) and *P. cyrtonema* polysaccharides (PCPs). Infrared (IR) spectroscopy and derivative spectral processing affirm both structural similarities and quantitative differences in functional groups among the species. Multivariate analyses, including HCA, PCA, and OPLS-DA, confidently classify the 12 batches of polysaccharides into three distinct groups (PSPs, PCPs, and PKPs), exhibiting strong model robustness (PCA: R^2^X = 0.951, Q^2^ = 0.673; OPLS-DA: R^2^Y = 0.953, Q^2^ = 0.922). Importantly, PKPs from number S11 show exceptional in vitro antioxidant activity (DPPH scavenging), which directly correlates with their high monosaccharide content and distinctive spectral features. These findings establish a robust foundation for the quality assessment of *Polygonatum* polysaccharides as potential natural antioxidants in functional foods, positioning PKPs as leading candidates for dietary supplement development.

## 1. Introduction

*Polygonatum* Mill. is a robust perennial plant known for its edible and medicinal properties, firmly belonging to the Liliaceae family. The 2025 edition of the *Chinese Pharmacopoeia* officially recognizes three primary source species: *P. kingianum*, *P. sibiricum*, and *P. cyrtonema*. These species are distinctly categorized on the market by their rhizome morphology: *P. cyrtonema* is identified as “ginger-shaped,” *P. sibiricum* as “chicken-head-shaped,” and *P. kingianum* as “plate-shaped” [1,2]. In traditional Chinese medicine (TCM), *Polygonatum* rhizomes are unequivocally recognized for their sweet flavor and neutral properties, explicitly targeting the spleen, lung, and kidney meridians. TCM theory asserts that sweet-tasting substances must not only provide sensory sweetness but also deliver significant functional benefits, such as tonic effects. Phytochemical studies consistently demonstrate that sweet herbs, including *Polygonatum*, are rich in saccharides, proteins, and amino acids—key components that underpin both their therapeutic effects in traditional medicine and functional food functionality as natural antioxidants, prebiotics, or texture modifiers [3]. Moreover, modern analyses have confirmed that polysaccharides serve as the principal bioactive components responsible for both the sweet flavor and the therapeutic effectiveness of *Polygonatum*, establishing them as definitive quality markers for this herb [4]. Extensive pharmacological research has substantiated that *Polygonatum* polysaccharides exhibit a range of bioactivity, including significant anti-inflammatory effects [5], robust immunomodulation function [6], strong antioxidant capacity [7], effective regulation of the gut microbiota [8], and outstanding neuroprotective properties [9].

The *Chinese Pharmacopoeia* (2025 edition) mandates that *Polygonatum* rhizomes contain at least 7.0% polysaccharides as a quality control standard [10]. However, this requirement is insufficient, as it ignores the critical monosaccharide composition and characteristic of *Polygonatum* polysaccharides [11]. This oversight leads to a quality assessment system that is fundamentally flawed and fails to represent the unique qualities of various *Polygonatum* species accurately.

The striking morphological and functional similarities between *Polygonatum* rhizomes and their closely related species, particularly *Polygonatum odoratum*, have led to significant confusion in clinical applications [12]. It is imperative to establish a more sensitive and specific quality control system to effectively enhance the authentication and safety evaluation of *Polygonatum* medicinal materials [13]. Previous studies have effectively characterized the polysaccharides of individual *Polygonatum* species, and demonstrated significant differences in their monosaccharide composition and molecular weight [9,14,15]. Thus, a comprehensive comparative analysis of the polysaccharides from the three most common *Polygonatum* spp. is challenging yet highly promising [10,16].

Traditional analytical techniques like high-performance liquid chromatography (HPLC) and gas chromatography (GC) are limited in terms of their focus on individual chemical markers. While they can provide preliminary fragment identification, they fail to capture the complete structural features of polysaccharides [17,18]. In stark contrast, infrared (IR) spectroscopy stands out as a highly effective analytical tool [19]. Its rapid, non-destructive, and high-throughput capabilities make it essential for global fingerprint profiling in complex matrix samples. Moreover, IR spectroscopy is crucial for the structural elucidation of complex natural products, particularly polysaccharides derived from TCMs. Meanwhile, analyzing correlations within the massive datasets of spectral information presents a substantial challenge worthy of attention. Fortunately, multivariate statistical analysis is a powerful solution capable of addressing this issue effectively [20]. The advantages of integrating GC and IR spectroscopy with techniques such as hierarchical cluster analysis (HCA), principal component analysis (PCA), and orthogonal partial least squares discriminant analysis (OPLS-DA) are substantial. However, the application of these methods in distinguishing Polygonatum polysaccharides has not been extensively explored and warrants further investigation.

Continuing our research on the quality control of traditional Chinese medicines [21,22,23,24], with a particular emphasis on the structure–activity relationships of *Polygonatum* polysaccharides [25,26,27,28,29,30], we herein are committed to (1) rigorously comparing the physicochemical properties, including monosaccharide composition and IR spectra, of polysaccharides from three distinct *Polygonatum* species; (2) developing a robust classification model through advanced multivariate statistical analysis; and (3) directly correlating the structural features of these polysaccharides with their antioxidant activity. By integrating second-derivative IR spectroscopy with chemometrics, the spectral resolution was significantly enhanced, and crucial discriminative peaks were pinpointed. Additionally, in line with the known antioxidant effects of *Polygonati* Rhizoma, a DPPH radical scavenging assay was conducted, confirming a significant link between structural differences and bioactivity.

Our findings firmly establish a scientific foundation for the quality control of *Polygonatum* polysaccharides, clearly highlighting *P. kingianum* as an excellent source of bioactive polysaccharides [15,31]. This study significantly enhances our understanding of the structure–activity relationships in *Polygonatum* polysaccharides [32] and provides a robust methodological framework for analyzing other complex natural products.

## 2. Materials and Methods

### 2.1. Materials and Chemicals

Twelve voucher specimens were deposited at the College of Chinese Medicine, Anhui University of Chinese Medicine (*Polygonatum sibiricum*—S1: Jinzhai, Anhui; S2: Danfeng, Shanxi; S3: Songxian, Luoyang; S4: Funiushan, Luoyang; *Polygonatum cyrtonema* Hua: S5: Zunyi, Guizhou; S6: Nanchong, Sichuan; S7: Puer, Yunnan; S8: Jiuhuashan, Chizhou. *Polygonatum kingianum*—S9: Yuxi, Yunnan; S10: Jinghong, Yunnan; S11: Puer, Yunnan in cultivated; S12: Puer, Yunnan in wild).

The chemicals utilized in this study were obtained from reputable suppliers, ensuring high-quality standards. We sourced trifluoroacetic acid (TFA), hydrogen peroxide, and 1,1-diphenyl-2-picrylhydrazyl (DPPH) from Shanghai Macklin Biochemical Co., Ltd. in Shanghai, China. Additionally, we acquired a comprehensive range of monosaccharides, including D-galacturonic acid (GalA), D-glucuronic acid (GlcA), D-ribose (Rib), L-rhamnose (Rha), L-arabinose (Ara), D-mannose (Man), D-galactose (Gal), D-xylose (Xyl), D-fructose (Fru), and D-glucose (Glc), from the National Institutes for Food and Drug Control in Beijing, China.

### 2.2. Preparation of Polysaccharides from Three Common Polygonatum Species

**Figure 1:**
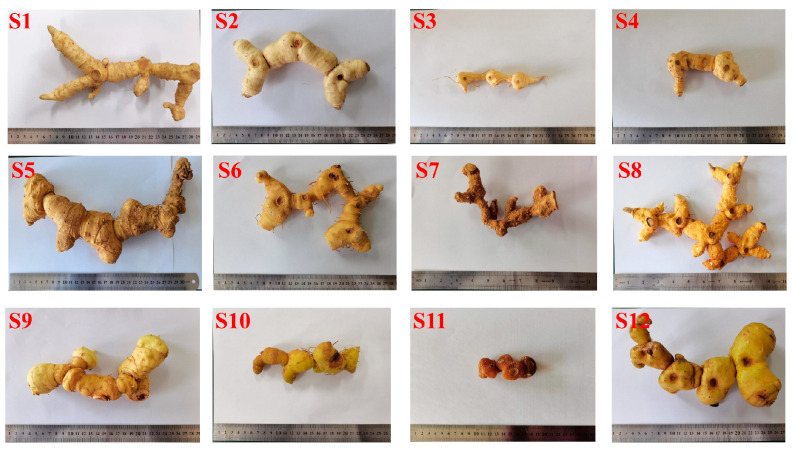
The 12 batches of rhizomes of three common *Polygonatum* spp. *(Polygonatum sibiricum*−S1: Jinzhai, Anhui; S2: Danfeng, Shanxi; S3: Songxian, Luoyang; S4: Funiushan, Luoyang. *Polygonatum cyrtonema* Hua−S5: Zunyi, Guizhou; S6: Nanchong, Sichuan; S7: Puer, Yunnan; S8: Jiuhuashan, Chizhou. *Polygonatum kingianum*−S9: Yuxi, Yunnan; S10: Jinghong, Yunnan; S11: Puer, Yunnan in cultivated; S12: Puer, Yunnan in wild).

The 12 batches of rhizomes of three common *Polygonatum* spp. (Figure 1) were extracted by boiling them in water at a 1:4 (*w*/*v*) ratio for 30 min. The crude polysaccharides were then precipitated using 80% ethanol, followed by protein removal via the Sevag method to purify the extract [33].

### 2.3. Monosaccharide Composition Analysis of Three Common Polygonatum spp. Polysaccharides

#### 2.3.1. Identification of Individual Chemical Markers by GC-MS

The monosaccharide composition of the hydrolyzed polysaccharide samples was effectively determined by GC-MS analysis. Specifically, 20 mg of polysaccharides were hydrolyzed using 2 mL of 2 mol/L trifluoroacetic acid (TFA) at 100 °C for 6 h. After hydrolysis, the solvent was evaporated under reduced pressure, and the dried hydrolysate was completely dissolved in 2 mL of anhydrous dimethyl sulfoxide (DMSO). Subsequently, 60 mg of NaOH powder was added, and the mixture was stirred at 35 °C for 30 min. Methylation was initiated by the careful addition of 1 mL of iodomethane (CH_3_I), allowing the reaction to proceed in a sealed vessel in the dark for 12 h. The reaction was then quenched with 2 mL of distilled water, and the methylated derivatives were extracted with dichloromethane for GC-MS analysis.

For comparison, monosaccharide standards, including glucuronic acid, galacturonic acid, mannose, xylose, galactose, glucose, fructose, arabinose, and rhamnose, were derivatized and analyzed under the same conditions.

The GC-MS analysis was performed under the same conditions as described in our previous report [29].

#### 2.3.2. Global Fingerprint Profiling by IR

##### Establishing Method

The polysaccharides (2 mg) were mixed with dried potassium bromide (200 mg), ground, and pressed in a vacuum. The IR spectrum of the polysaccharides was acquired using a Nicolet 5700 FT-IR spectrometer (Thermo Fisher, Waltham, MA, USA) in the wavelength region of 4000–500 cm^−1^.

##### Method Validation

The precision of our method was rigorously evaluated through intra-day and inter-day variability tests. For the intra-day assessment, the standard solution was analyzed six times within a single day, demonstrating our commitment to consistency. Inter-day variability was established by repeating the analysis over three consecutive days. We ensured sample stability by testing extracts at room temperature at intervals of 0, 1, 2, 3, 4, and 5 h. Spiking the samples with known quantities of standard compounds enabled us to effectively conduct recovery studies. All results were thoroughly assessed using relative standard deviation (RSD), confirming the reliability of our findings.

### 2.4. In Vitro Antioxidant Activity

The antioxidant activity of the samples was evaluated using DPPH radical scavenging assays. The DPPH radical scavenging capacity of polysaccharides from three species of *Polygonatum* was rigorously evaluated using a modified method from the literature [34]. We prepared a fresh DPPH solution (0.2 mM in methanol, 2.0 mL) and combined it with 2.0 mL of polysaccharide solutions at varying concentrations (2–10 mg/mL). The reaction mixtures were vortexed and incubated at 25 °C for 30 min in darkness. Absorbance was subsequently measured at 524 nm using a UV-2550 spectrophotometer (Shimadzu Corporation, Kyoto, Japan). The scavenging activity was calculated as follows:

$$\mathrm{DPPH}\;\mathrm{radical}\;\mathrm{scavenging}\;\mathrm{rate}\;(\%)=\;\left(1-\frac{A_{sample}-A_{control}}{A_{blank}}\right)\times 100\%$$

where A_blank_ is the absorption of the blank sample, A_sample_ is the absorption of the analytical sample, and A_control_ is the absorption of the background (where the DPPH solution was replaced with distilled water).

### 2.5. HCA, PCA, and OPLS-DA

Statistical analyses were decisively conducted using R software 4.3.0 version (https://www.r-project.org, accessed on 15 April 2025). We undertook multivariate data treatment on 30 × 11 matrices derived from the infrared spectra of 12 batches of *Polygonati* Rhizoma polysaccharides. Our analysis utilized intergroup mean associations based on the relative peak heights of 9 common IR spectral peaks as the foundational data. Sample similarity was effectively calculated using angle cosine distance metrics, allowing for the systematic clustering of polysaccharides from three distinct species of *Polygonatum*. Additionally, data standardization was rigorously performed in SIMCA version 14.1, with principal components selected based on their eigenvalues and contribution rates [35].

### 2.6. Statistical Analysis

All graphical representations were generated using GraphPad Prism 7.0 and Origin 8.0. Data analysis was conducted with SPSS 23.0, employing one-way ANOVA followed by Student’s *t*-test for pairwise comparisons. All experiments were carried out in triplicate, and the results are presented as the mean ± standard deviation (SD). We set statistical significance at *p* < 0.05.

In this study, DPPH clearance data were expressed as the mean ± 95% confidence interval (CI). The confidence interval was calculated using the t-distribution, and the formula is

confidence interval=x¯±ta2,df×s√n

where $\bar{x}$ is the mean value, is the standard deviation, *n* = 5 (5 concentration gradients), the degree of freedom df = *n* − 1 = 4, and for a 95% confidence level, t_0.025,__4_ = 2.776. The data were processed using SPSS 23.0 software.

## 3. Results and Discussion

### 3.1. Comprehensive Evaluation of PSPs, PCPs, and PKPs

#### Monosaccharide Composition

**Figure 2:**
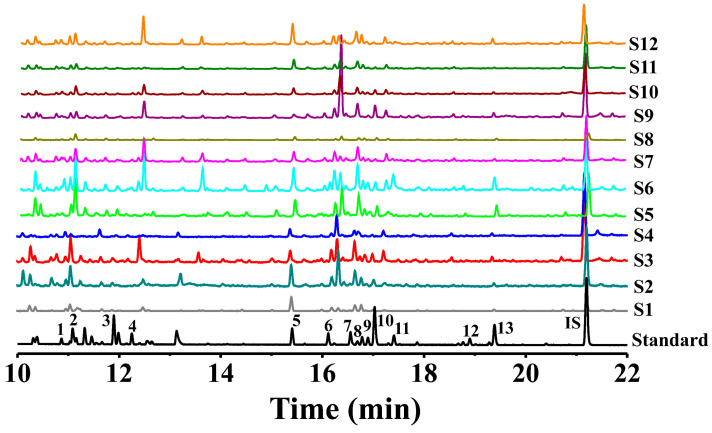
Monosaccharide composition and quantitative analysis of three common *Polygonatum* polysaccharides. (1: Xyl, 10.87 min; 2: Rha, 11.16 min; 3: Rib, 11.98 min; 4: Ara, 12.26 min; 5: β-D-Glc, 15.41 min; 6: Fru, 16.11 min; 7: α-D-Man, 16.55 min; 8: α-D-Glc, 16.79 min; 9: α-D-Gal, 16.88 min; 10: β-D-Man, 17.02 min; 11: β-D-Gal, 17.39 min; 12: GlcA, 18.90 min; 13: GalA, 19.39 min; IS: 19.68 min).

The monosaccharide composition analysis results for the polysaccharides extracted from three common *Polygonatum* spp. are illustrated in Figure 2. By comparing the peak retention times with those of standard monosaccharides, it was observed that the four batches of *P. sibiricum* polysaccharides (PSPs) (S1–S4) contained Glc, Man, Xyl, GalA, and GlcA. S1 and S2 also contained Gal, while S3 and S4 did not. Notably, the Glc content in the polysaccharides of *P. sibiricum* from S2 was higher than that from other regions. Concerning the four batches of *P. cyrtonema* polysaccharides (PCPs) (S5–S8), they primarily consisted of Glc, Man, Gal, GalA, and GlcA. In comparison, the polysaccharides from S8 did not contain Xyl, and the Glc content in PCPs from S6 was higher than in other regions. Similarly, the four batches of *P. kingianum* polysaccharides (PKPs) (S9–S12) were mainly composed of Glc, Man, Gal, Xyl, GalA, and GlcA. The glucose content of PKPs from S11 was notably higher than in other regions. Moreover, upon comparing the monosaccharide types and content in polysaccharides from the three common *Polygonatum* spp., it was evident that PKPs had significantly higher levels than PSPs and PCPs. These distinct monosaccharide profiles provide a basis for the targeted design of ingredients in dietary supplements. For example, the fructan structure of *P. cyrtonema* polysaccharides may function as a prebiotic to modulate the gut microbiota in functional foods.

### 3.2. Effects of PCPs, PSPs, and PKPs on Antioxidant Activity

**Figure 3:**
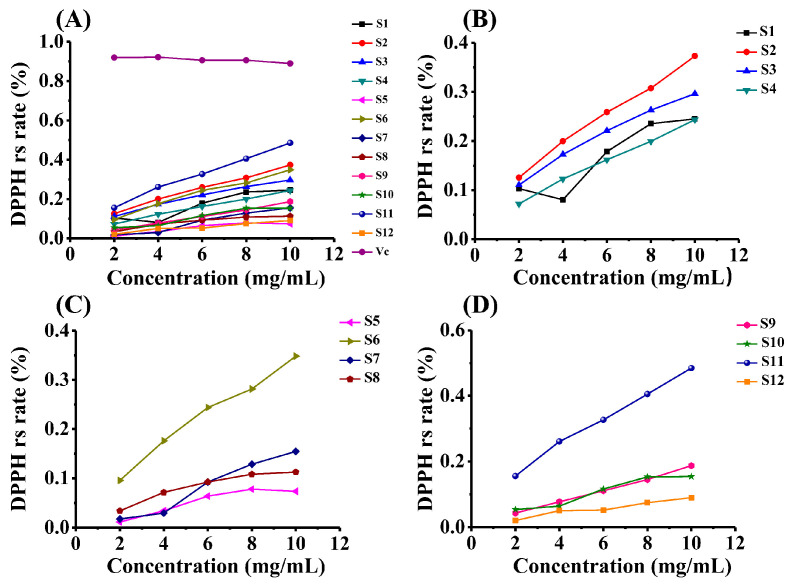
DPPH radical scavenging capacity of polysaccharides from three *Polygonatum* species. (**A**) Comparative antioxidant activity among species; (**B**) *P. sibiricum* polysaccharides (PSPs); (**C**) *P. cyrtonema* polysaccharides (PCPs); (**D**) *P. kingianum* polysaccharides (PKPs).

The DPPH radical scavenging assay was selected as a standard method for preliminary antioxidant evaluation, given its wide acceptance in natural product research [7,36]. This method efficiently reflects the hydrogen-donating capacity of polysaccharides, which is directly linked to their hydroxyl-rich structures (evidenced by IR spectroscopy at 3409 cm^−1^) and monosaccharide composition (e.g., glucose content in PKPs). Polysaccharides extracted from TCM possess remarkable radical scavenging properties, attributable primarily to their unique molecular structures [37]. This distinctive composition underscores their potential as powerful natural antioxidants. Herein, the results of the DPPH scavenging activity for polysaccharides derived from three common *Polygonatum* spp. are presented in Figure 3. Notably, the DPPH scavenging activity of PKPs from S11 exhibited the highest potency among all samples, comparable to synthetic antioxidants (e.g., BHT). This activity supports its potential as a natural preservative in food systems, inhibiting lipid oxidation. Furthermore, the antioxidant activity of PSPs from S2 surpassed that of other PSPs, while the PCPs from S6 demonstrated more potent antioxidant activity than other PCPs. Overall, the antioxidant potential of the PKPs from S11 was the most robust among the various samples analyzed.

The DPPH clearance rates of different *Polygonatum* samples showed significant differences. As a positive control, the mean value of Vc (0.908248) was significantly higher than that of all *Polygonatum* samples, and the 95% confidence interval (0.892340~0.924156) was the narrowest, indicating that the activity stability was the best. In contrast, the S8 sample had the lowest mean (0.052144) and a wide confidence interval (0.016793~0.087495), suggesting that its clearance rate fluctuated greatly. The confidence interval width of Vc was only 0.031816, which was significantly narrower than that of all *Polygonatum* samples, indicating that its DPPH clearance exhibits excellent repeatability, thereby verifying the reliability of the experimental method. The detailed statistical results of each sample are shown in the Table 1 below.

### 3.3. Quantitative Analysis for Polysaccharides from Three Common Polygonatum spp.

#### 3.3.1. Method Validation

The precision of the proposed method was rigorously evaluated through eight consecutive injections, allowing for the calculation of the relative standard deviation (RSD) for the polysaccharides, which was determined to be 0.40%. The stability of the method was also assessed by conducting five injections immediately after mixing and another set of injections 5 h later. The results demonstrated an RSD of 0.31%, underscoring the robust reproducibility and reliability of the proposed method.

#### 3.3.2. IR Spectrum Analysis

**Figure 4:**
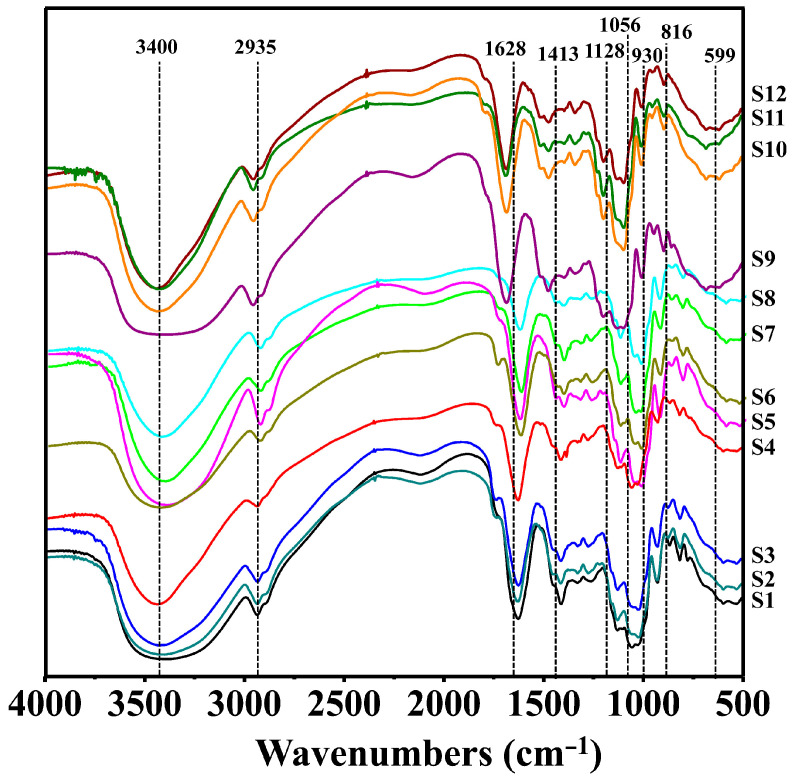
IR fingerprints of three kinds of polysaccharides from three common *Polygonatum* spp. *(Polygonatum sibiricum*−S1: Jinzhai, Anhui; S2: Danfeng, Shanxi; S3: Songxian, Luoyang; S4: Funiushan, Luoyang. *Polygonatum cyrtonema* Hua−S5: Zunyi, Guizhou; S6: Nanchong, Sichuan; S7: Puer, Yunnan; S8: Jiuhuashan, Chizhou. *Polygonatum kingianum*−S9: Yuxi, Yunnan; S10: Jinghong, Yunnan; S11: Puer, Yunnan in cultivated; S12: Puer, Yunnan in wild).

The IR spectrum fingerprint analysis of the polysaccharides derived from three common *Polygonatum* spp. revealed strikingly similar peak shapes and positions within the range of 4000–400 cm^−1^. However, slight variations were observed in the peak heights (light transmittance). These results clearly show that polysaccharides from various *Polygonatum* species exhibit distinct functional group vibrations, despite variations in their relative abundances. The average infrared spectrum derived from three species (refer to Figure 4) reveals three crucial absorption bands: a broad and intense hydroxyl stretching vibration at 3409 cm^−1^, a weak aliphatic C-H stretch at 2930 cm^−1^, and a prominent carbonyl stretching band at 1630 cm^−1^ [38]. Additionally, the absorption peak at 1406 cm^−1^ indicates the symmetric bending vibration of a methyl group, while the absorption zone ranging from 1200 to 950 cm^−1^ mainly represents mixed vibrations of the polysaccharide [36]. Within the range of 950 to 700 cm^−1^, characteristic absorptions are primarily related to sugar ring skeleton vibrations [39]. These spectral features definitively confirm the presence of typical polysaccharide structures and underscore the subtle structural differences among the species.

#### 3.3.3. IR Spectrum Analysis

**Figure 5:**
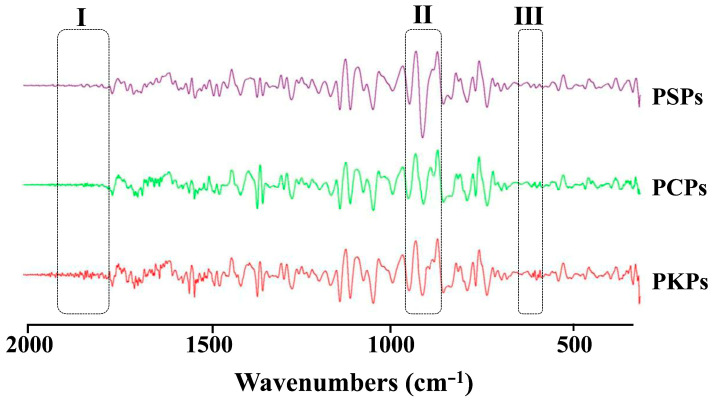
Average spectra of IR second derivatives of polysaccharides from PSPs, PCPs, and PKPs.

The derivative method is widely utilized for baseline correction and background elimination in spectroscopic analysis. By converting the original spectrum into first and second derivatives, constant and linear backgrounds can be effectively removed, respectively [40]. This conversion results in a spectrum with more pronounced features, enhancing the resolution of the infrared spectrum and revealing hidden, overlapped peaks. In this study, the infrared spectral data of polysaccharides from three common *Polygonatum* spp. were processed using the second derivative method based on overlapping spectra. As illustrated in Figure 5, the absorption peaks of the polysaccharides from three common *Polygonatum* spp. exhibited variations within the 2000–400 cm^−1^ range. Subsequently, second derivative processing was performed within this range. In region I, the number and intensity of absorption peaks for PKPs were notably higher than those for PSPs and PCPs. A distinctive derivative absorption peak was observed for the polysaccharides from three common *Polygonatum* spp. in region II, albeit with differing peak intensities. The absorption peak intensity of PSPs was significantly higher than that of PCPs and PKPs. Region III displayed two absorption peaks for PSPs, whereas both PCPs and PKPs had four absorption peaks. Consequently, based on the distinctive features of the derivative absorption peaks observed in these three regions, effective differentiation of polysaccharides from three common *Polygonatum* spp. was achieved.

#### 3.3.4. HCA

**Figure 6:**
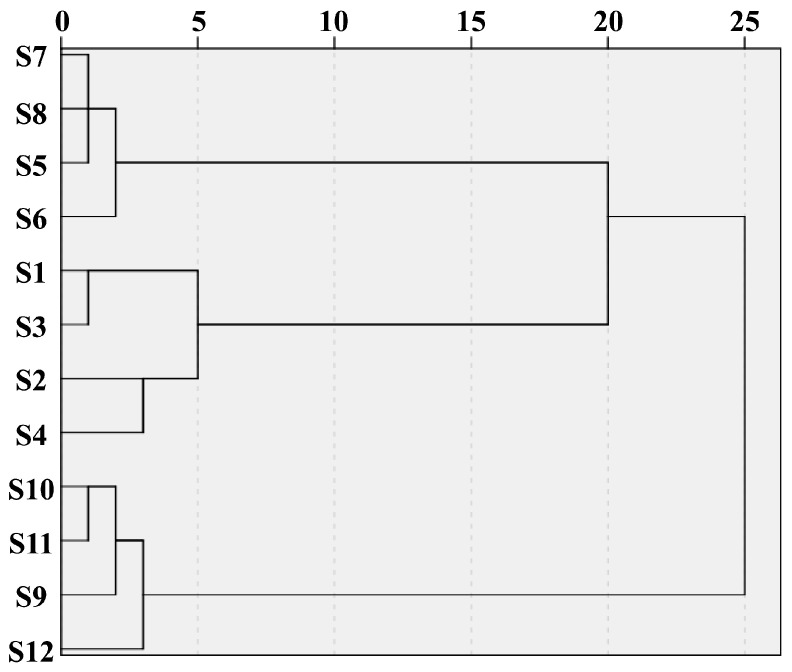
The HCA of the polysaccharides from three common *Polygonatum* spp. *(Polygonatum sibiricum*−S1: Jinzhai, Anhui; S2: Danfeng, Shanxi; S3: Songxian, Luoyang; S4: Funiushan, Luoyang. *Polygonatum cyrtonema* Hua−S5: Zunyi, Guizhou; S6: Nanchong, Sichuan; S7: Puer, Yunnan; S8: Jiuhuashan, Chizhou. *Polygonatum kingianum*−S9: Yuxi, Yunnan; S10: Jinghong, Yunnan; S11: Puer, Yunnan in cultivated; S12: Puer, Yunnan in wild).

The OMNIC 8.2 and IBM SPSS Statistics 26.0 software packages were used to process infrared spectrum data. Ward’s method and Euclidean distance were used for hierarchical cluster analysis (HCA). As shown in Figure 6, when the discriminant distance was 25, the 12 batches of the polysaccharides from three common *Polygonatum* spp. could be clustered into two groups: four batches of PKPs (S9–S12) in Group I, and four batches of PSPs (S1–S4) and PCPs (S5–S9) in Group II. The eight batches of samples could be further classified into two groups, among which the PSPs (S1–S4) were classified as Group IIa and the PCPs (S5–S8) were classified as Group IIb. The above results indicate that the infrared spectra of the polysaccharides from three common *Polygonatum* spp. were different.

#### 3.3.5. PCA

**Figure 7:**
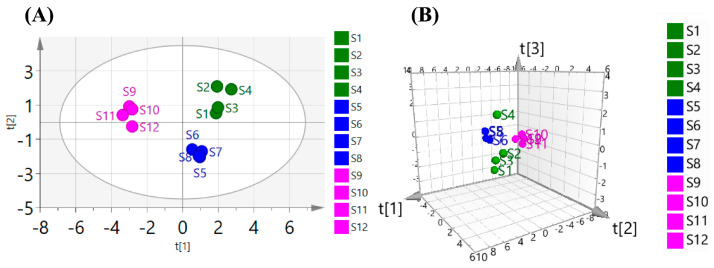
The PCA of the polysaccharides from three common *Polygonatum* spp. (**A**) 2D; (**B**) 3D. *(Polygonatum sibiricum*−S1: Jinzhai, Anhui; S2: Danfeng, Shanxi; S3: Songxian, Luoyang; S4: Funiushan, Luoyang. *Polygonatum cyrtonema* Hua−S5: Zunyi, Guizhou; S6: Nanchong, Sichuan; S7: Puer, Yunnan; S8: Jiuhuashan, Chizhou. *Polygonatum kingianum*−S9: Yuxi, Yunnan; S10: Jinghong, Yunnan; S11: Puer, Yunnan in cultivated; S12: Puer, Yunnan in wild).

PCA is a powerful statistical technique that effectively reduces the dimensionality of data. It transforms correlated variables into a smaller set of uncorrelated principal components, all while maintaining the critical information from the original dataset. It has the advantages of simplicity and accuracy in fingerprint research. Therefore, to better objectively reflect the differences among the polysaccharides from three common *Polygonatum* spp., the relative peak heights of nine typical peaks in the infrared spectrum of 12 batches of *Polygonatum* spp. polysaccharides were taken as the original data. The SPSS 26.0 and SIMCA 14.1 software packages were used for principal component analysis. The model quality parameters were R^2^X = 0.951 and Q^2^ = 0.673. In multivariate modeling, R^2^X (goodness of fit) decisively quantifies the cumulative proportion of variance explained in the X-matrix, demonstrating how effectively the model reproduces the original data. Q^2^ (goodness of prediction) provides a clear assessment of the model’s predictive capability through rigorous cross-validation. Both metrics are essential, ranging from 0 to 1, and values approaching 1.0 unequivocally indicate outstanding model performance in terms of both explanation and prediction accuracy. R^2^X and Q^2^ were greater than 0.5, indicating good stability and predictive power of the model. Based on this, a three-dimensional score diagram of the principal component analysis was created, as shown in Figure 7. The 12 batches of *Polygonatum* spp. polysaccharides could be roughly divided into three categories: S1–S4 were PSPs, S5–S8 were PCPs, and S9–S12 were PKPs.

In addition, the data were suitable for factor analysis (KMO = 0.694; Bartlett’s χ^2^ = 96.606, *p* < 0.001), indicating a significant correlation between the variables. Both results show that the data were applied to factor analysis. The characteristic value and variance contribution rate of principal components are shown in Table 2.

Taking the characteristic value greater than one as the extraction standard, two principal components, 1 and 2, were obtained, with a cumulative variance contribution rate of 83.492%. This can represent the essential characteristics and primary information of the nine typical peaks in 12 batches of *Polygonatum* spp. polysaccharides, so the first two factors could be used as the principal components. According to the two principal component coefficients, SPSS 26.0 software was used to calculate the principal component scores of 12 batches of *Polygonatum* spp. polysaccharides, and the linear combination of y1 and y2 was obtained.

y1 = −0.373 × Zf1 + 0.396 × Zf2 + 0.356 × Zf3−0.387 × Zf4 + 0.206 × Zf5 + 0.246 × Zf6 + 0.376 × Zf7 − 0.206 × Zf8 + 0.374 × Zf9

y2 = 0.012 × Zf1 − 0.235 × Zf2 + 0.335 × Zf3 + 0.244 × Zf4+0.555 × Zf5 + 0.444 × Zf6 + 0.146 × Zf7 + 0.406 × Zf8 + 0.285 × Zf9

The formula for calculating the comprehensive score was y = 0.5864 × y1 + 0.2485 × y2. The comprehensive scores of the main components of 12 batches of *Polygonatum* spp. polysaccharides were arranged in descending order. The higher the comprehensive score, the better the quality of the medicine was. As shown in Table 3, the comprehensive score of the main components of PKPs was the highest, followed by PSPs, and the lowest score was obtained for PCPs. S11 in four batches of PKPs had the highest score, S2 in four batches of PSPs had the highest score, and S6 in four batches of PCPs had the highest score, respectively.

#### 3.3.6. OPLS-DA

**Figure 8:**
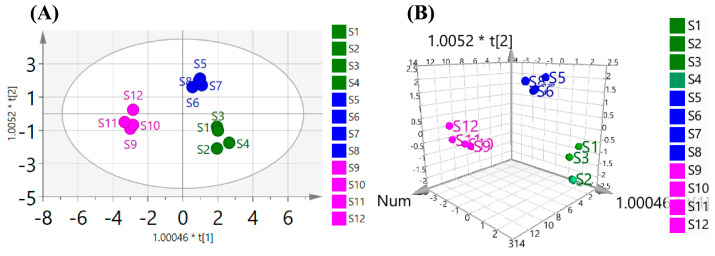
The OPLS-DA analysis of three common *Polygonatum* spp. polysaccharides. (**A**) 2D; (**B**) 3D. *(Polygonatum sibiricum*−S1: Jinzhai, Anhui; S2: Danfeng, Shanxi; S3: Songxian, Luoyang; S4: Funiushan, Luoyang. *Polygonatum cyrtonema* Hua−S5: Zunyi, Guizhou; S6: Nanchong, Sichuan; S7: Puer, Yunnan; S8: Jiuhuashan, Chizhou. *Polygonatum kingianum*−S9: Yuxi, Yunnan; S10: Jinghong, Yunnan; S11: Puer, Yunnan in cultivated; S12: Puer, Yunnan in wild).

OPLS-DA is a highly effective supervised analysis method, particularly useful for addressing classification and discrimination challenges. In this study, OPLS-DA was employed to further examine the distinctions among the 12 batches of *Polygonatum* spp. polysaccharides, building upon the insights from PCA. The relevant model parameters for the OPLS-DA analysis were as follows: R^2^X = 0.834, R^2^Y = 0.953, and Q^2^ = 0.922. These values, akin to those in PCA, signify the goodness of fit and predictive ability of the model, with R^2^Y reflecting the cumulative interpretation rate in the *Y*-axis direction, which indicates the stability and quality of cross-validation predictions. In Figure 8, the OPLS-DA results illustrate excellent intra-group aggregation and inter-group classification of the 12 batches of *Polygonatum* spp. polysaccharides. No overlap was observed among different samples, indicating a superior classification effect compared to the PCA score chart. Notably, the PSPs, PCPs, and PKPs were distinctly clustered, affirming the effectiveness of OPLS-DA in distinguishing the polysaccharides from three common *Polygonatum* spp. These results align with the findings from PCA and HCA.

## 4. Conclusions

In conclusion, we establish an integrated approach to effectively distinguish three medicinal Polygonatum species through comprehensive polysaccharide characterization. By combining the GC-MS technique and IR with multivariate statistical methods, we identify significant discrepancies in the structural and functional properties of PCPs, PSPs, and PKPs. P. kingianum shows a significantly higher glucose content. The structural differences correlate directly with functional variations, particularly the PKPs of number S11, which exhibit remarkable antioxidant activity in DPPH radical scavenging assays. The superiority of this antioxidant is attributed to its unique monosaccharide composition and specific spectral features. This research addresses gaps in current quality control standards by moving beyond simple polysaccharide quantification to comprehensive characterization. The integration of spectroscopic fingerprinting, chemometrics, and bioactivity evaluation establishes a robust framework for evaluating other medicinal polysaccharides. The proposed method serves as a robust preliminary tool for quality control. At the same time, future research will integrate FRAP, ABTS, and cellular assays to explore broader bioactivity aligned with the herb’s traditional uses. The integration of spectroscopic fingerprinting, chemometrics, and bioactivity evaluation establishes a robust framework for evaluating other medicinal polysaccharides. Furthermore, this study significantly enhances our understanding of the relationship between *Polygonatum* polysaccharides and food–medicine homology, providing a methodological framework for analyzing functional food ingredients. The identified structure–activity relationships support the development of *Polygonatum*-derived antioxidant supplements and natural food preservatives.

## Figures and Tables

**Table 1:** Table showing 95% confidence intervals of DPPH clearance rates of 12 batches of *Polygonatum sibiricum* calculated based on t-distribution.

Sample	Mean Value	95% Confidence Interval (Lower Limit~Upper Limit)
S1	0.15076	0.117888~0.183632
S2	0.112358	0.042594~0.182122
S3	0.326436	0.168262~0.484610
S4	0.10799	0.048957~0.167023
S5	0.22898	0.108162~0.349798
S6	0.083622	0.009179~0.158065
S7	0.084396	0.009953~0.158839
S8	0.052144	0.016793~0.087495
S9	0.168506	0.075545~0.261467
S10	0.212622	0.121317~0.303927
S11	0.159772	0.077289~0.242255
S12	0.252788	0.134187~0.371389
VC	0.908248	0.892340~0.924156

**Table 2:** Characteristic value and variance contribution rate of each component.

Component	Initial Eigenvalue			Load Sum of Squares		
	Characteristic Value	Variance Contribution Rate (%)	Cumulative Variance Contribution Rate (%)	Characteristic Value	Variance Contribution Rate (%)	Cumulative Variance Contribution Rate (%)
1	5.278	58.643	58.643	5.278	58.643	58.643
2	2.236	24.849	83.492	2.236	24.849	83.492
3	0.683	7.591	91.083			
4	0.361	4.013	95.096			
5	0.208	2.313	97.409			
6	0.137	1.523	98.932			
7	0.055	0.610	99.542			
8	0.025	0.279	99.820			
9	0.016	0.180	100.000			

**Table 3:** The ranking of PCA.

No.	Component 1	Component 2	Component 3
S11	3.37	0.42	2.08
S9	2.97	0.94	1.97
S10	2.85	0.75	1.86
S12	2.82	−0.25	1.59
S2	−1.93	2.10	−0.61
S6	−0.55	−1.62	−0.72
S3	−1.97	0.88	−0.94
S1	−1.88	0.56	−0.96
S8	−0.91	−2.01	−1.03
S7	−1.10	−1.69	−1.07
S5	−0.96	−2.03	−1.07
S4	−2.71	1.95	−1.11

## Data Availability

The original contributions presented in the study are included in the article, further inquiries can be directed to the corresponding authors.
